# Distributed Optical Fiber Sensing of Temperature Rise During 110 kV Conductor–Ground Wire Ice-Shedding Discharge

**DOI:** 10.3390/mi17010032

**Published:** 2025-12-27

**Authors:** Yanpeng Hao, Zijian Wu, Lei Huang, Yashuang Zheng, Qi Yang, Yao Zhong, Huan Huang

**Affiliations:** 1 School of Electric Power, South China University of Technology, Guangzhou 510640, Chinaepzjwu@mail.scut.edu.cn (Z.W.);; 2 Electric Power Research Institute of Guizhou Grid Co., Ltd., Guiyang 550000, China

**Keywords:** ice-shedding discharge, discharge monitoring, distributed optical fiber sensing, Brillouin frequency shift, OPGW

## Abstract

Ice-shedding on overhead transmission lines can easily lead to jump discharge and subsequent line tripping, and effective monitoring methods are still lacking. To address this problem, this study proposes a distributed optical fiber sensing approach based on Brillouin optical time-domain reflectometry (BOTDR) for detecting ice-shedding discharge on 110 kV conductor–ground wire. The optical fibers embedded in an optical fiber composite overhead ground wire (OPGW) are used as sensing elements. Through simulated ice-shedding discharge experiments under different icing conditions, the Brillouin frequency shift (BFS) characteristics along the OPGW fiber are investigated, and the relationship between the BFS increment caused by the discharge-induced temperature rise and the discharge parameters is revealed. The experimental results show that ice-shedding discharge produces a localized temperature-rise region in the OPGW fiber, with an axial extent of 20–40 cm and a duration of 2–4 s. The maximum BFS increment due to the discharge temperature rise, Δ*v_T_*_m_, is strongly dependent on the icing condition. Under conditions of no icing, light rime, and glaze ice on the conductor only, Δ*v_T_*_m_ remains within 5.43–7.94 MHz, whereas when both the conductor and ground wire are covered with glaze ice, Δ*v_T_*_m_ decreases significantly to 2.91–3.76 MHz. Further analysis indicates that, to satisfy the requirements for detecting ice-shedding discharge, the BOTDR must achieve a spatial resolution better than 0.1 m and a temporal sampling rate of no less than 5 Hz. These findings verify the feasibility of using distributed optical fiber sensing technology to detect ice-shedding discharge and provide experimental support for studies on the associated discharge mechanisms.

## 1. Introduction

Ice accretion on overhead transmission lines and the resulting ice-shedding have become major threats to the safe and stable operation of power grids, especially as the construction of UHV projects in China shifts toward the southwestern regions [1,2]. Ice-shedding refers to the dynamic process in which ice on the surfaces of conductors and ground wires detaches either uniformly or non-uniformly under environmental factors such as rising temperature and wind excitation, causing large-amplitude, low-frequency galloping of the line span [3]. This process leads to a rapid reduction of the air gap between phases. When the gap insulation strength becomes lower than the operating voltage, flashover and line tripping may occur. Statistics show that ice-shedding has become the leading cause of ice-related tripping events on transmission lines. For example, from 2009 to 2017, more than 60% of ice-related faults in the Xinjiang power grid were attributed to ice-shedding [4]. Given the high frequency and severity of such events, real-time monitoring and mechanism analysis of ice-shedding discharge are of significant engineering value for improving ice-disaster prevention and mitigation capabilities.

Existing studies on ice-shedding primarily focused on the dynamic response of transmission lines under different ice-shedding conditions and on predicting jump height. In early physical experiments, Morgan et al. conducted ice-shedding tests on a five-span 132 kV line in 1964 and measured the maximum jump height after ice detachment in the mid-span [5]. In 2011, Meng et al. used a full-scale multi-span test platform and applied concentrated masses to simulate ice load, thereby obtaining the dynamic characteristics of conductor displacement and tension [6]. In 2023, Huang et al. developed a scaled conductor model that satisfies geometric, material, and dynamic similarity criteria and systematically analyzed the effects of bundle number, span length, elevation difference, initial tension, and ice thickness on the dynamic response [7,8]. In numerical studies, Fekr et al. used ADINA software in 1998 to perform nonlinear dynamic simulations of two-span conductors under 21 typical scenarios involving different ice thicknesses, spans, elevation differences, and ice-shedding locations [9]. In 2018, Wang established a multi-span, three-degree-of-freedom model and revealed the influence of random, non-uniform ice-shedding conditions—including the shedding location, removal rate, and shedding frequency—on jump characteristics [10]. Based on these findings, simplified engineering formulas for predicting jump height were proposed by Wen [11] and Zhang [12], providing theoretical support for safety assessments and accident back-analysis during transmission-line design. However, these approaches are predictive in nature and do not offer real-time monitoring of the ice-shedding process.

Research on monitoring techniques for ice-shedding is relatively limited. In 2014, Huang et al. used inertial measurement units to obtain the conductor acceleration and angular velocity, then reconstructed the galloping trajectory via motion reconstruction algorithms [13]. In 2016, Huang reconstructed jump trajectories using binocular stereo vision; however, image acquisition devices installed on towers are susceptible to occlusion and may fail under severe ice and snow conditions [14]. In 2020, Feng established a strain-transfer model for fiber Bragg grating composite insulators and measured the vertical and horizontal dynamic loads during ice-shedding, with peak-to-valley stress deviations within 30% of those from tension sensors [15]. Although these methods can monitor local dynamic responses, they require additional structural modifications and cannot achieve long-distance, distributed measurements.

Distributed fiber optic sensing (DFOS) uses optical fibers simultaneously as sensing media and signal transmission channels, enabling full spatiotemporal monitoring along transmission lines. DFOS offers a wide measurement range, a high spatial resolution, and strong electromagnetic immunity [16,17] and has been preliminarily applied in monitoring conductor icing. In 2025, Hao et al. proposed a BOTDR-based method for determining the minimum critical sensing thickness of natural ice accretion on loose-tube OPGW and achieved decoupling of BOTDR temperature–strain cross sensitivity using micro-meteorological temperature data [18,19]. In the same year, Dong et al. established the relationship between the optical phase and conductor strain for composite fiber-core phase conductors and proposed a phase-sensitive OTDR-based method for locating ice-shedding, determining shedding rates, and measuring jump heights [20]. However, existing DFOS techniques mainly monitor mechanical responses and cannot determine whether discharge faults occur during ice-shedding. Ice-shedding discharge involves both mechanical strain changes due to dynamic axial stress and localized temperature rises caused by arc heating and discharge Joule heating. Specifically, air gap discharge exhibits significant photo-thermal effects, and advanced techniques such as spectral pyrometry have been successfully applied to analyze temperature characteristics under unknown emissivity conditions [21]. Studies in [22,23] measured the temperature-rise characteristics of lightning strikes on OPGW using DFOS, achieving lightning-point localization and energy estimation and providing a potential technical path for monitoring ice-shedding discharge based on fiber temperature. Distinct from traditional mechanical monitoring of line galloping and DFOS-based lightning detection, ice-shedding discharge sensing requires the synchronous measurement of both “jump” and “discharge” events. This involves a unique mechanothermal coupling mechanism where localized thermal signatures must be extracted from a high-dynamic-strain background, dictating specific spatial and temporal resolution requirements.

To address these challenges, this study constructs a distributed optical fiber monitoring system for 110 kV conductor–ground wire ice-shedding discharge at a natural icing field test site. A BOTDR-based method is proposed to monitor temperature rise in OPGW fibers during ice-shedding discharge, and discharge detection and localization are achieved by decoupling mechanical strain and thermal effects. Experiments under four typical icing conditions—no icing, light rime, glaze ice, and the conductor and ground wire covered with glaze ice—are conducted to investigate the relationship between the Brillouin frequency shift (BFS) increment caused by OPGW temperature rise and the discharge current amplitude and maximum discharge distance. The results verify the feasibility of using DFOS to detect ice-shedding discharge, reveal the influence mechanism of icing conditions on temperature-rise sensing characteristics, and provide experimental support for discharge-mechanism studies.

## 2. Experimental Principle

### 2.1. Brillouin Optical Time-Domain Reflectometry

BOTDR enables distributed sensing based on the stimulated Brillouin scattering effect in optical fibers. Thermally excited acoustic waves inside the fiber induce periodic variations in density, resulting in a traveling-wave modulation of the refractive index. When a probe pulse is launched into the fiber, this periodic refractive index modulation produces Brillouin scattering, and the scattered light exhibits a Brillouin frequency shift with respect to the incident light. The BFS is mainly affected by fiber temperature and strain variations [16] and can be expressed as

(1) $$\nu (T,\varepsilon )=v(T_{0},\varepsilon_{0})+\Delta \nu_{T}+\Delta \nu_{\varepsilon }$$

where *v*(*T*, *ε*) is the BFS under temperature *T* and strain *ε*; *v*(*T*_0_, *ε*_0_) is the BFS at the initial temperature *T*_0_ and initial strain *ε*_0_; and Δ*v_T_* and Δ*v_ε_* are the BFS increments induced by temperature and strain changes, respectively. Based on the time-domain reflectometry principle, the forward propagation of the optical pulse continuously generates backward Brillouin scattering. By locating the time delay of the backscattered signal, the temperature and strain distribution along the fiber can be obtained.

**Figure 1:**
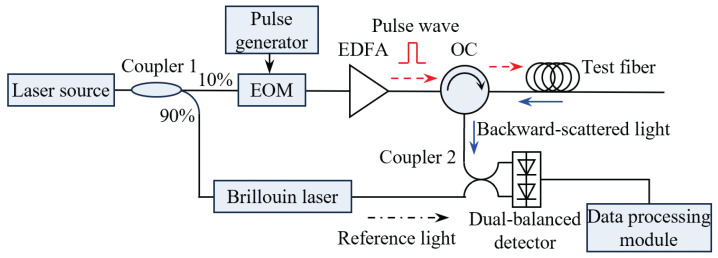
Self-heterodyne coherent-detection BOTDR configuration.

Since the spontaneous Brillouin scattering signal is extremely weak (typically 10^−9^ of the incident optical power), BOTDR employs self-heterodyne coherent detection to improve the signal-to-noise ratio. The system configuration is shown in Figure 1. A narrow-linewidth laser is divided by a 10/90 polarization-maintaining coupler (Coupler 1). The 10% portion serves as the probe light, which is modulated into pulses by an electro-optic modulator (EOM) driven by a pulse generator, amplified by an erbium-doped fiber amplifier (EDFA), and injected into the test fiber via an optical circulator (OC). The remaining 90% acts as the local oscillator and is frequency-shifted by a Brillouin laser. The backward-scattered light from the test fiber is extracted by the OC and combined with the reference light by Coupler 2. A dual-balanced detector receives the combined signal and outputs the intermediate-frequency beat signal, and the BFS distribution along the fiber is demodulated by the data processing module.

### 2.2. OPGW Sensing Characteristics and Monitoring Mechanism for Ice-Shedding Discharge

OPGW features high mechanical strength, strong anti-torsion capability, and a compact structure, making it suitable for transmission lines in heavy-icing regions [18]. The OPGW consists of single-mode communication fibers housed in a metal protection tube and an outer layer of aluminum-clad steel strands. When the internal fiber is connected to the BOTDR, the OPGW serves simultaneously as a sensing element and a communication channel, enabling real-time monitoring of transmission-line operating conditions.

In practical transmission lines, the ground wire (including OPGW) is installed higher than the phase conductors and carries no current; thus, it experiences heavier ice loading and is more prone to ice-shedding. When ice-shedding of the OPGW triggers inter-phase discharge, the BFS in the fiber is influenced by two coupled mechanisms:

(1) Ice-shedding strain: Axial stress variations induced by the jump propagate through the aluminum-clad steel strands and the metal tube, eventually reaching the encapsulated fiber. The fiber is subjected to rapid dynamic tensile strain, causing changes in the BFS.
(2) Discharge temperature rise: Gap breakdown produces a high-temperature arc (typically 7000–8000 °C [24]), and both arc heating and Joule heating of the discharge current heat the metal layers of the OPGW through thermal radiation and conduction. The heat is further transferred into the internal fiber, causing a local temperature rise.

**Figure 2:**
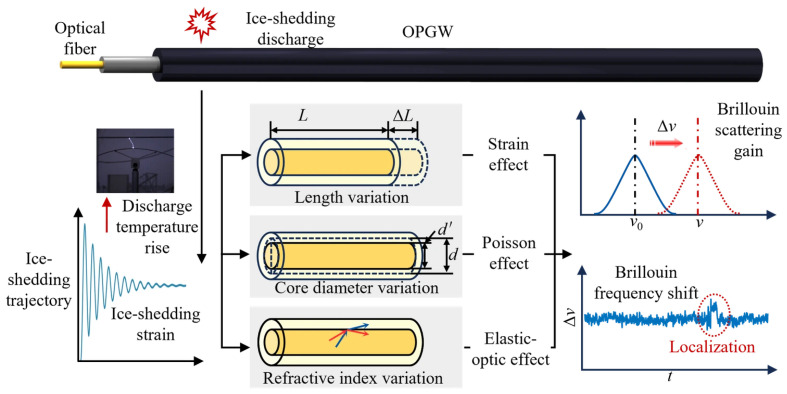
Physical mechanism of BFS variation induced by ice-shedding discharge in OPGW.

These two effects jointly alter the geometric and optical properties of the fiber—such as its length, core diameter, and refractive index—resulting in strain, Poisson, and elasto-optic effects and ultimately leading to changes in the BFS, as shown in Figure 2. According to Equation (1), decoupling the mechanical-strain-induced and temperature-rise-induced BFS increments enables the identification and localization of ice-shedding discharge.

## 3. Experimental System and Methods

### 3.1. Experimental System

In a previous study [24], a simulated 110 kV conductor–ground wire ice-shedding discharge test platform was constructed at the natural icing field base of the China Southern Power Grid Disaster Prevention and Reduction Joint Laboratory, and related experiments were carried out under typical natural icing conditions. By building on this platform, a distributed optical fiber sensing system for ice-shedding discharge detection was further developed in this work.

**Figure 3:**
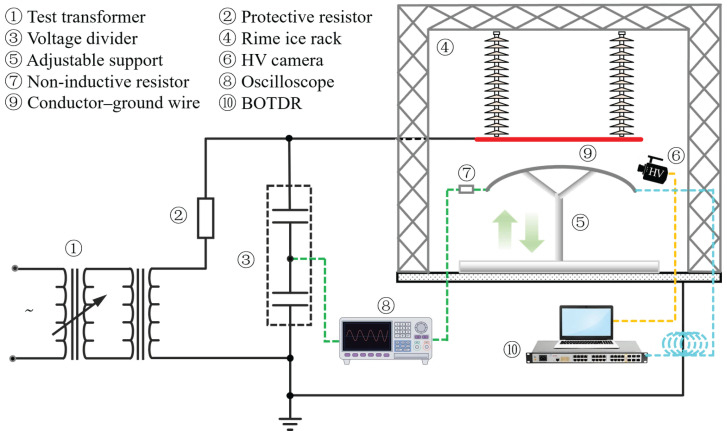
Distributed optical fiber sensing system for detecting ice-shedding discharge on 110 kV conductor–ground wire.

The experimental system is shown in Figure 3. Since this study focused on ice-shedding discharge physics rather than structural dynamics, both the conductor and ground wire were represented by simplified short specimens. The simulated conductor consisted of a 3 m long homogeneous steel tube with a 20 mm outer diameter, closely matching the dimensions of actual LGJ-185/45 conductors (19.6 mm diameter) used in 110 kV lines. The simulated ground wire was an OPGW-24B1-100 cable of the same length as the conductor, featuring a stranded stainless-steel tube structure with a diameter of 13.2 mm and embedded G.652D single-mode fibers. The OPGW fiber under test was fusion-spliced to the BOTDR device via a fiber jumper.

The simulated conductor was suspended below a rime ice rack using composite insulators, while the simulated ground wire was mounted on an insulated adjustable support equipped with a servo motor. By controlling the vertical motion of the support, the ice-shedding process was reproduced, and the displacement control accuracy was ±0.2 mm. The system operating voltage was set to the phase voltage of the 110 kV system (63.5 kV). The method for determining the maximum discharge distance (*d*_m_) during ice-shedding is detailed in [25].

During the tests, visible arc images, the discharge current, and the BFS of the fiber were recorded simultaneously. Arc images were captured using a Phantom high-speed camera at 4000 fps. The discharge current was obtained from the voltage across a 1 Ω non-inductive resistor in the grounding circuit, measured by a Tektronix MDO3024 oscilloscope with a sampling rate of 2.5 GS/s. The BFS of the OPGW fiber was monitored in real time using a high-dynamic-range BOTDR, the main technical specifications of which are listed in Table 1. Under a 100 m measurement range, the BOTDR provides a sampling rate of 100 Hz and a spatial resolution of 0.1 m, meeting the rapid-response requirements for ice-shedding discharge. All measurement devices were synchronized using a unified timing system, and electrical, optical, and BFS signals were recorded through timestamp-based synchronization.

### 3.2. Icing Conditions

This study focused on four typical icing conditions under which ice-shedding discharge may occur: (1) no icing; (2) light rime with a thickness of 2 mm; (3) glaze ice with a thickness of 15 mm; (4) glaze ice of 15 mm on the conductor only.

**Figure 4:**
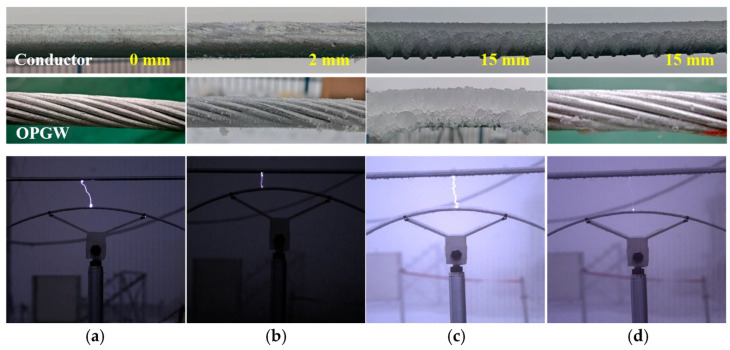
Ice coverage on the conductor–ground wire and corresponding arc images at the maximum discharge distance: (**a**) no icing; (**b**) light rime with a thickness of 2 mm; (**c**) glaze ice with a thickness of 15 mm; (**d**) glaze ice of 15 mm on the conductor only.

For each condition, 3–5 repeated tests were conducted during continuous low-temperature periods. The results are summarized in Table 2. The maximum discharge distance *d*_m_ was 15 cm under no icing, 8–9 cm under light rime, 17 cm under glaze ice, and 18–19 cm when the conductor was covered with glaze ice while the ground wire was ice-free. Good consistency was observed among repeated tests, with a maximum deviation of only 1 cm. Representative icing morphologies and arc images captured at the instant of *d*_m_ are shown in Figure 4.

### 3.3. Decoupling Method for Temperature-Rise-Induced BFS

To extract the Brillouin features associated with the temperature rise during ice-shedding discharge, a comparative experimental method was adopted. Two test groups were designed:

(1) Ice-shedding group: Only the mechanical motion of ice-shedding was applied, without voltage. The resulting BFS variation was recorded as Δ*v*_1_(*T*, *ε*).
(2) Ice-shedding discharge group: Under identical mechanical motion conditions, the operating voltage was applied, causing breakdown across the gap. The resulting frequency-shift variation was recorded as Δ*v*_2_(*T*, *ε*).

The two groups of tests were performed within the same time window, and the lift parameters of the support frame were kept identical (displacement accuracy ± 0.2 mm), ensuring comparable mechanical strain. Therefore, the BFS increment induced solely by discharge-generated temperature rise can be expressed as

(2) $$\Delta \nu_{T}=v_{2}(T,\varepsilon )-v_{1}(T,\varepsilon )$$

In this relation, the strain-induced components approximately cancel, assuming that the discharge-generated mechanical shock is negligible compared with ice-shedding strain. Therefore, Δ*v_T_* represents the BFS caused only by the discharge-induced temperature rise.

## 4. Results and Discussion

### 4.1. Characteristics of the BFS Along the Optical Fiber

**Figure 5:**
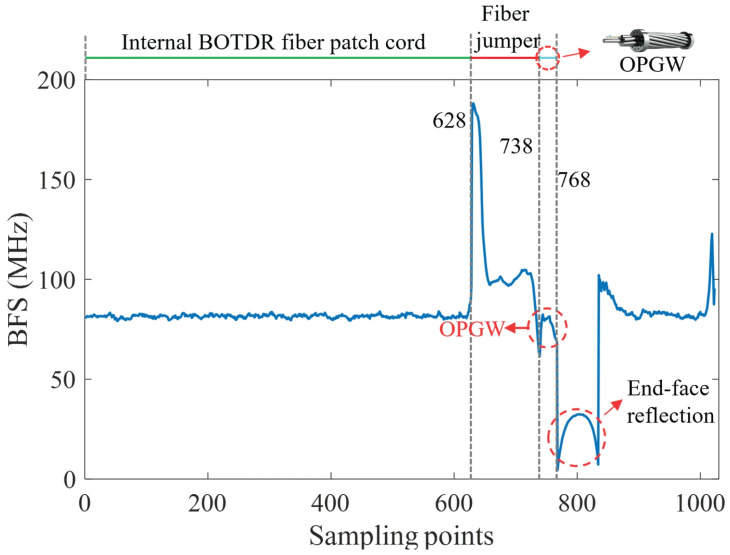
The distributed BFS measured along the optical fiber by BOTDR.

In this study, the maximum one-way measurement range of the BOTDR was configured to 100 m, corresponding to 1024 sampling points and a spatial resolution of approximately 0.1 m. Figure 5 illustrates the BFS distribution along the fiber at a representative moment. The overall BFS profile exhibits a distinct segmented pattern, corresponding directly to the different fiber sections within the optical path.

Sampling points 1–628 correspond to the internal fiber patch cord inside the BOTDR, where the BFS remains stable at approximately 80 MHz. Sampling points 629–738 represent the external fiber jumper. A noticeable BFS discontinuity occurs at the splice between the internal and external fibers, after which the BFS stabilizes around 100 MHz. Sampling points 739–768 correspond to the OPGW-embedded sensing fiber. In this region, the BFS shows slight spatial fluctuations due to OPGW structural bending, non-uniform mechanical stress, and the relatively short sample length. Signals recorded beyond sampling point 769 originate from the end-face reflection echo, which exhibits a markedly different amplitude from the preceding Brillouin backscattering signal, allowing clear identification of the fiber termination.

### 4.2. Spatiotemporal Distribution of BFS During Ice-Shedding Discharge

**Figure 6:**
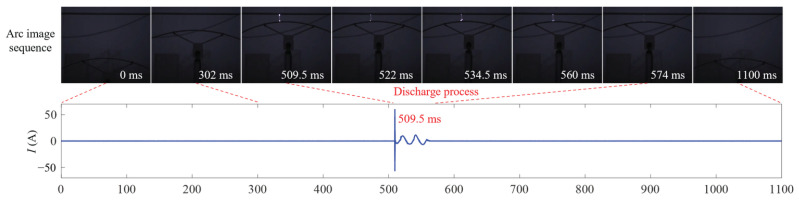
Visible arc images and discharge current during ice-shedding discharge under light rime condition (*d*_m_ = 8 cm).

Taking the light rime condition with a maximum discharge distance of *d*_m_ = 8 cm as an example, Figure 6 shows the typical temporal evolution of the ice-shedding discharge, captured simultaneously through visible arc imaging and discharge current (*I*) measurement. The moment when the ground wire begins to move is defined as *t* = 0 ms. As shown in Figure 6, as the gap between the conductor and ground wire gradually decreases and approaches *d*_m_, an arc first forms at approximately 509.5 ms, instantly bridging the gap. At that instant, the discharge current transitions rapidly from a weak corona current to a breakdown pulse with a peak value of 60.5 A, then decays within about 0.5 ms into a power-frequency follow current. During the follow current stage, both the arc luminosity and current waveform exhibit periodic variations due to the zero-crossings of the AC voltage. The arc is eventually extinguished following the reclosing operation of the line. The total arc duration is approximately 64 ms, with the significant discharge current persisting for about 50 ms.

**Figure 7:**
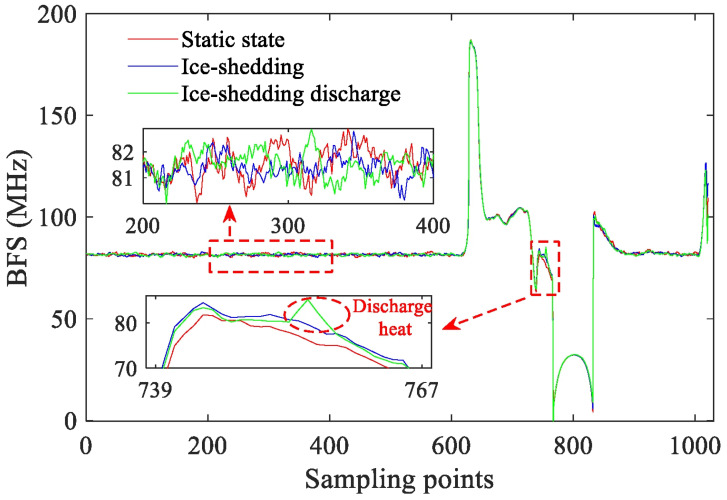
A comparison of the BFS distributions along the optical fiber under three conditions: static, ice-shedding, and ice-shedding discharge.

Figure 7 compares the BFS distributions along the fiber under three conditions: conductor–ground wire static, ice-shedding (only mechanical), and ice-shedding discharge. The BFS variations in both the internal BOTDR fiber and the external jumper are small, with differences less than 2 MHz. In contrast, the BFS of the OPGW-embedded fiber shows notable differences among the three states. Compared with the static condition, the BFS exhibits a 24 MHz variation during ice-shedding due to mechanical strain; during ice-shedding discharge, the BFS further increases and forms a localized peak several megahertz higher at the discharge point. This peak is caused by the local temperature rise induced by the arc discharge.

**Figure 8:**
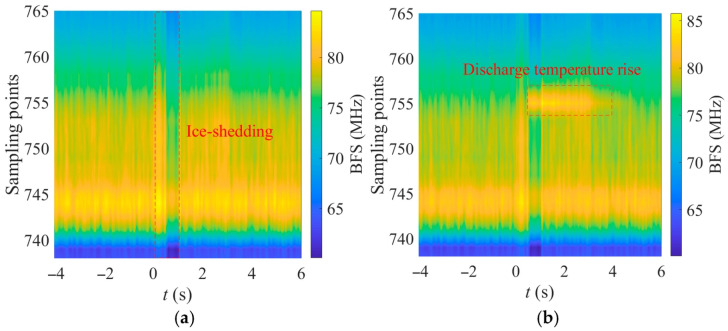
Spatiotemporal evolution of the BFS along the OPGW fiber during (**a**) ice-shedding and (**b**) ice-shedding discharge.

For further analysis of the thermal characteristics, Figure 8 shows the spatiotemporal BFS evolution along the OPGW fiber during de-icing galloping and ice-shedding discharge. At *t* = 0 ms, the mechanical movement induces a sudden change in axial stress inside the OPGW, causing the BFS along the embedded fiber to exhibit an evolution pattern of “increase–decrease–recovery” during the entire motion period (0–1.1 s), with slight differences among sampling points. Compared with the purely mechanical response shown in Figure 8a, the discharge-induced temperature rise in Figure 8b causes an additional BFS increase near the discharge point, forming a temperature-rise region with significantly larger amplitude than the strain background. According to the visible arc images, the discharge point is located slightly to the left of the OPGW center; based on the BOTDR monitoring range (sampling points 739–768), the temperature-rise region corresponds to sampling points 754–757, which agrees well with the actual discharge location. This confirms that BFS variation can effectively locate the ice-shedding discharge.

**Figure 9:**
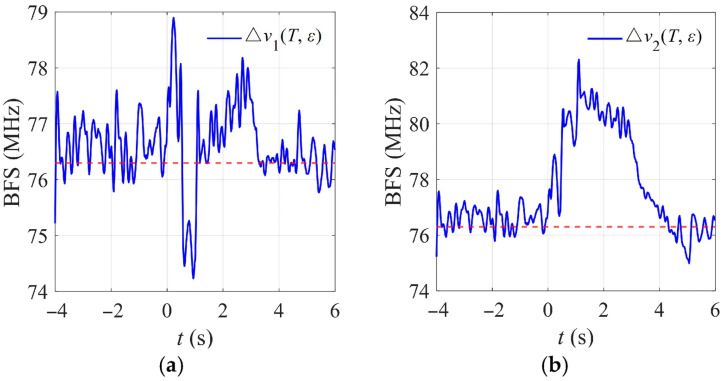
Temporal evolution of the BFS at sampling point 756 under (**a**) ice-shedding and (**b**) ice-shedding discharge.

Taking sampling point 756 within the temperature rise as an example, its temporal BFS evolution is shown in Figure 9. Under static conditions, the BFS remains at approximately 76.3 MHz, represented by the red dashed line, with fluctuations within 2 MHz due to system noise. After the onset of mechanical movement, the BFS first increases to about 78.8 MHz, then decreases to approximately 74.2 MHz during the strain release stage and gradually returns to the noise level after the motion ends. When the discharge-induced temperature rise is superimposed, the maximum BFS at this sampling point reaches approximately 82.3 MHz, and it returns to the static level about 4 s after the discharge ends.

### 4.3. BFS Increment Induced by Temperature Rise During Ice-Shedding Discharge

**Figure 10:**
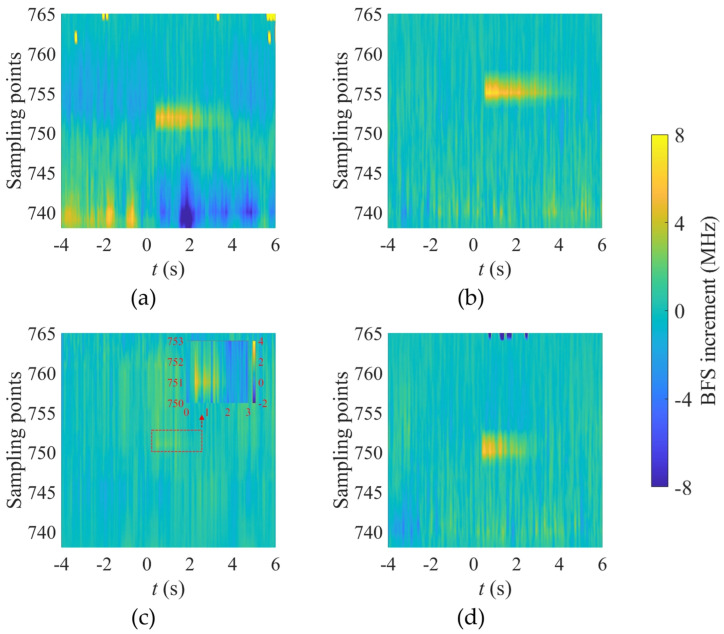
Spatiotemporal distributions of the OPGW BFS increment *Δv_T_* under different icing conditions: (**a**) no icing; (**b**) light rime; (**c**) glaze ice; (**d**) glaze ice on the conductor only.

Figure 10 presents the typical spatiotemporal distributions of the BFS increment *Δv_T_* of the OPGW under different icing conditions. The statistical results show that the axial extent of the temperature-rise region is approximately 20–40 cm, and its duration is about 2–4 s. According to the Nyquist sampling criterion [26], the spatial sampling intervals must be smaller than half of the minimum scale of the target event. Therefore, given the measurement range settings, the spatial resolution of the BOTDR should be better than 0.1 m to ensure at least two valid sampling points within the temperature-rise region. Such sampling density allows the formation of a distinguishable local BFS peak, enabling accurate discharge localization. Furthermore, to reliably identify transient events in the time domain—especially to distinguish the temperature-rise process from system noise—a temporal sampling rate of no less than 5 Hz is recommended. This ensures that no fewer than ten sampling points are obtained at a single location during the temperature-rise event, guaranteeing detection reliability.

The maximum temperature-rise-induced BFS increment is denoted by Δ*v_T_*_m_. Table 2 summarizes the maximum discharge distance *d*_m_, discharge current amplitude *I*_m_, and corresponding Δ*v_T_*_m_ for 110 kV conductor–ground wire ice-shedding under different icing conditions. The results indicate a clear negative correlation between *d*_m_ and *I*_m_ under operating voltage—that is, a smaller *d*_m_ corresponds to a larger *I*_m_. However, no similar correlation is observed between *d*_m_ and Δ*v_T_*_m_ across different icing conditions. Specifically, for the conditions of no ice, light rime, and glaze ice of 15 mm on the conductor only, Δ*v_T_*_m_ remains within 5.43–7.94 MHz, despite differences in *d*_m_. In contrast, when both the conductor and ground wire are covered with glaze ice—i.e., a thicker ice layer exists at the discharge point—Δ*v_T_*_m_ drops significantly, falling within 2.91–3.76 MHz, approximately 3 MHz lower than in other conditions.

The temperature rise generated by ice-shedding discharge is primarily attributed to the combined effect of heat transfer from the high-temperature arc and Joule heating from the transient breakdown current. The arc temperature is sustained by thermionic emission, whereas the Joule heat depends on both the peak value and the duration of the breakdown current. Although the peak discharge current can reach several tens of amperes, its duration is extremely short (on the millisecond scale), and, thus, the contribution of Joule heating to the overall temperature rise is relatively limited. Based on the experimental results, it can be inferred that when the discharge point is covered by a thick ice layer, the heat released by the arc must penetrate the ice layer before reaching the OPGW embedded fiber. The ice layer increases the thermal resistance of the heat conduction path, suppressing heat transfer to the fiber core and resulting in a significantly reduced temperature rise; consequently, Δ*v_T_*_m_ becomes markedly smaller than that under lighter icing conditions.

In summary, the icing condition at the discharge point directly affects the magnitude of the OPGW temperature-rise signature and is one of the key factors limiting the capability of distributed optical fiber sensing technology to reliably detect ice-shedding discharge events.

## 5. Conclusions

A distributed optical fiber sensing system for monitoring ice-shedding discharge was established at an outdoor test site. Discharge experiments under various icing conditions were conducted, and the BFS characteristics along the OPGW-embedded fiber were analyzed. The main conclusions are as follows:

(1) A distributed detection method for 110 kV conductor–ground wire ice-shedding discharge based on OPGW-embedded fiber was proposed. By using the OPGW internal fiber as the sensing element and conducting simulated ice-shedding experiments, this study demonstrated that the transient temperature rise induced by the discharge led to a significant increase in BFS, and that the differences in BFS characteristics between temperature rise and mechanical strain were clearly identifiable under the experimental conditions.

(2) The relationship between discharge parameters and the BFS increment caused by fiber temperature rise was revealed. Under different icing conditions, the maximum discharge distance *d*_m_ and the discharge current amplitude *I*_m_ exhibited a negative correlation. In contrast, the maximum temperature-rise-induced BFS increment Δ*v_T_*_m_ showed no correlation with *d*_m_ or *I*_m_. For the conditions of no ice, light rime, and a conductor with glaze ice while the ground wire was ice-free, Δ*v_T_*_m_ remained stable within 5.43–7.94 MHz. When both the conductor and ground wire were covered with glaze ice, Δ*v_T_*_m_ decreased to 2.91–3.76 MHz, approximately 3 MHz lower than in other cases. The presence of an ice layer at the discharge point introduced significant thermal resistance to heat transfer into the fiber, thereby reducing the detectable temperature rise and limiting the sensitivity of BFS-based temperature detection.

(3) Key BOTDR configuration parameters for monitoring OPGW ice-shedding discharge were proposed. Experimental results showed that the axial extent of the temperature-rise region was approximately 20–40 cm, with a duration of 2–4 s. For reliable detection and localization of the discharge event, it is recommended that the BOTDR achieve a spatial resolution better than 0.1 m and a temporal sampling rate of no less than 5 Hz for monitoring OPGW under 110 kV conditions.

This work verified a new technical approach for identifying ice-shedding discharge by distinguishing localized BFS increments Δ*v_T_* from the large-scale mechanical strain background. By quantifying the necessary spatial resolution (<0.1 m) and sampling rate (≥5 Hz), it provides a crucial experimental foundation for applying distributed optical fiber sensing to the electrical safety monitoring of transmission lines. Future research will explore the integration of multi-mechanism sensing technologies, such as ROTDR and φ-OTDR, leveraging multiple fibers within the OPGW to further enhance decoupling performance and monitoring reliability under complex environmental conditions, including non-uniform icing and variations in ice quality.

## Figures and Tables

**Table 1:** Key parameters of the high-dynamic-range BOTDR.

No.	Parameter	Specification
1	Maximum measurement range	≤2.5 km
2	Single-shot measurement time	0.25 s @ 2.5 km
3	Spatial resolution	≤1 m @ 2.5 km
4	Strain measurement accuracy	±5 με

**Table 2:** Statistics of *d*_m_, *I*_m_, and Δ*v_T_*_m_ for 110 kV conductor–ground wire ice-shedding under different icing conditions.

Icing Conditions	*d*_m_ (cm)	*I*_m_ (A)	Δ*v_T_*_m_ (MHz)
No ice	15	44.5	6.55
	15	42.1	7.23
	15	43.2	5.43
	15	41.6	6.12
Light rime	8	60.5	6.80
	8	57.6	7.94
	9	53.0	5.77
	8	59.3	—
	8	56.1	—
Glaze ice	17	38.3	3.76
	17	38.0	2.91
	17	38.3	3.45
Glaze ice on conductor only	19	34.4	6.43
	18	37.6	6.12
	19	33.9	6.73
	19	32.8	—

## Data Availability

The original contributions presented in this study are included in the article. Further inquiries can be directed to the corresponding author.
